# Exploring Nanogeochemical Environments: New Insights from Single Particle ICP-TOFMS and AF4-ICPMS

**DOI:** 10.1021/acsearthspacechem.1c00350

**Published:** 2022-04-04

**Authors:** Manuel D. Montaño, Chad W. Cuss, Haley M. Holliday, Muhammad B. Javed, William Shotyk, Kathryn L. Sobocinski, Thilo Hofmann, Frank von der Kammer, James F. Ranville

**Affiliations:** † Department of Environmental Sciences, Western Washington University, Bellingham, Washington 98225, United States; ‡ Department of Renewable Resources, University of Alberta, Edmonton T6G 2H1, Alberta, Canada; § School of Science and the Environment, Grenfell Campus, Memorial University of Newfoundland, Corner Brook A2H 5G4, Newfoundland, Canada; ∥ Department of Chemistry, Western Washington University, Bellingham, Washington 98225, United States; ⊥ Sciences Department of Environmental Geosciences, Centre for Microbiology and Environmental Systems Science, University of Vienna, Althanstrasse 14, Vienna 1090, Austria; # Department of Chemistry, Colorado School of Mines, Golden, Colorado 80401, United States

**Keywords:** Single particle ICP-MS, Field flow fractionation, nanoparticles, nanogeoscience, ICP-TOFMS

## Abstract

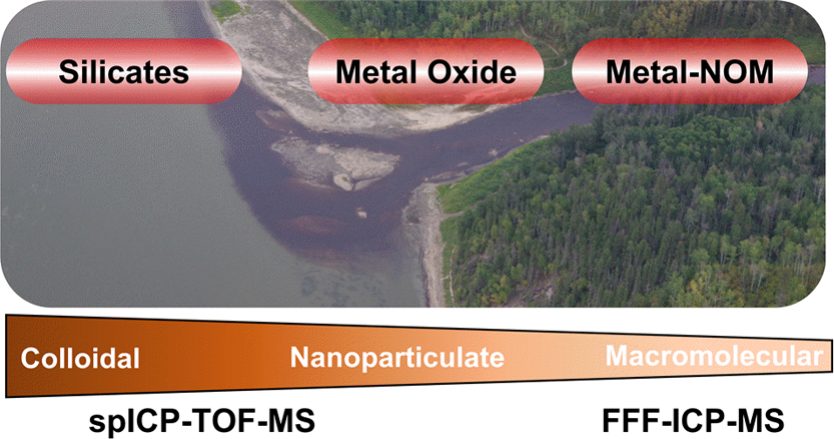

Nanogeochemistry is an emerging focus area recognizing the role of nanoparticles in Earth systems. Engineered nanotechnology has cultivated advanced analytical techniques that are also applicable to nanogeochemistry. Single particle inductively coupled plasma ICP-time-of-flight-mass spectrometry (ICP-TOF-MS) promises a significant step forward, as time-of-flight mass analyzers enable simultaneous quantification of the entire atomic mass spectrum (∼7–250 *m*/*z*^+^). To demonstrate the utility of this approach, samples were collected and analyzed from a large, boreal river, and its surrounding tributaries. These samples provided us with a diversity of particle compositions and morphologies, while their interconnected nature allowed for an examination of the various nanogeochemical processes present in this system. To further expand on this effort, we combined this high-throughput technique with AF4-ICPMS, focusing on major carriers of trace elements. Using spICP-TOF-MS, Al, Si, and Fe were grouped into classes having all combinations of one or more of these elements. Particle-by-particle ICP-TOF-MS analysis found chemically heterogeneous populations, indicating the predominance of diverse mineralogy or heteroaggregates. The importance of suspended Fe and Mn for the speciation of Pb was observed by single particle ICP-TOF-MS and complemented by AF4-ICPMS analysis of dissolved organic matter and nanoparticulate Fe/Mn. Our study exploits the combination of spICP-TOF-MS and AF4-ICP-MS for studying isotopic and elemental ratios (mineralogy) of individual nanoparticles, which opens the door to further explore the mechanisms of colloid facilitated transport of trace elements.

## Introduction

Particles with diameters <5 μm are major transport vectors for trace elements (TEs) and pollutants in natural aquatic systems.^1,2^ Colloids (0.001–1 μm) and especially nanoparticles (0.001–0.1 μm) are most important for many aspects of biogeochemistry and pollutant transport and fate due to their greater surface area-to-mass ratio, higher number concentrations, and tendency to remain suspended.^3−5^ Smaller particles are also more relevant in ecotoxicology, as they can adsorb a greater quantity of potentially toxic contaminants per unit of mass.^6,7^ Recognizing that nanoparticles play a key role in many natural and human-altered geochemical cycles has led to the addition of Nanogeoscience to the Earth sciences.^8−10^ While larger particles adsorb less contaminant per unit of mass, their origin as weathering products may facilitate the connection of composition to source and formation processes.

Fluxes of nanoparticles, colloids, and micron-sized particles are dynamic in large boreal rivers like the Athabasca River (AR, Alberta, Canada).^11,12^ Snowmelt and storms mobilize and suspend large quantities of particles, while variation in catchment properties and associated TE sources between the AR and its tributaries lead to corresponding variation in particle properties. Given their high sensitivity to climate change, improved understanding of boreal river systems is needed.^13^ The transport of TEs by suspended sediments in rivers has been the subject of study for many decades;^14,15^ however, the lack of effective analytical tools has limited investigation of the nanogeochemical environment and associated processes.

Measuring the abundance and properties of aquatic particles is challenging due to their small size, polydispersity, chemical heterogeneity, and variable colloidal stability.^16−18^ Measuring particle size and composition typically relies on physical separation to distinguish hydrologically suspended (>1 μm), chemically dispersed (0.001–1 μm), and solvated (ionic) forms. However, the physical separation of particles and corresponding binary classification of TEs as either dissolved (often <0.45 μm) or particulate (>0.45 μm), both obscure the importance of particles in the filtrate and create artefacts that disturb the in situ particle size distribution. Subsequent analysis also relies on low-throughput and operationally challenging techniques such as electron microscopy, light scattering, or elemental analysis.^19−21^ The nanotechnology revolution has produced new analytical tools to assess the environmental risks of engineered nanoparticles (ENPs), which are also useful for naturally occurring nanoparticles (NNPs).^22^

Recent developments in single particle inductively coupled plasma-time-of-flight-mass spectrometry (spICP-TOFMS) have been driven by the need for more complete characterizations of ENPs.^23−25^ Simultaneous multi-element detection by spICP-TOFMS provides characterization and quantification of multiple elements in individual particles across the isotope mass spectrum.^26^ However, few studies have examined the ability of spICP-TOFMS to conduct nanogeochemical analyses in aquatic systems by analyzing the composition of NNPs and natural colloids.^27^

Although powerful, spICP-TOF-MS cannot generally observe nanoparticles <20–50 nm, with some elements having size detection limits >100 nm.^28^ A complimentary element-specific approach is therefore needed to examine the entire nanogeochemical environment. In this study, asymmetric flow field-flow fractionation (AF4) coupled to ICP–MS (AF4-ICP-MS) was applied to 0.45 μm filtered samples to quantify TEs associated with inorganic phases and macromolecules (e.g., DOC) less than 450 nm. Regions of the AF4-ICP-MS fractogram (detector response vs elution time) were combined into three categories: macromolecular, nanoparticulate, and colloidal. Assignments were made based on both size (elution time) and detector response: absorbance at a wavelength of 254 nm (*A*_254_) for organic matter and ICP–MS for inorganic phases. When used with spICP-TOFMS, AF4-ICPMS facilitates analysis of the complete nanogeochemical environment. Herein, we demonstrate the effectiveness and potential of these combined techniques as a frontier approach for examining the nanogeochemical environment of river systems, via application to the AR and its tributaries.

## Methods and Materials

### Sample Characteristics

**Figure 1:**
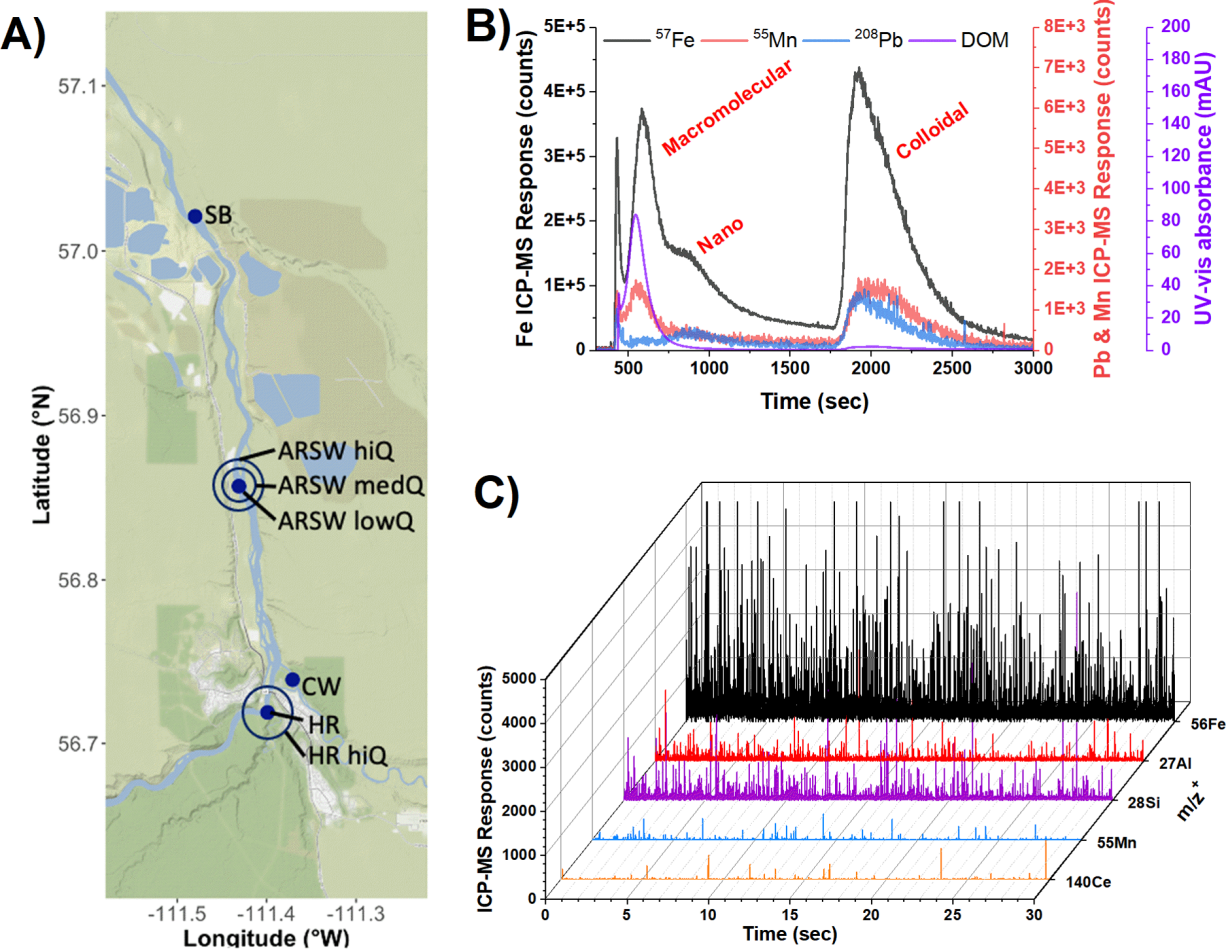
Sampling sites and analytical approaches to monitor particle populations in the AR: (A) Selected sample sites for the AR at different flow rates (hiQ, medQ, and lowQ) and its tributaries (Clearwater River = “CW”, Steepbank River = “SB”, Horse River = “HR”); (B) AF4-ICPMS fractogram detailing different particle fractions; (C) spICP-TOF-MS time plot showing multiple particle events with overlapping elemental signals.

Samples were selected from a larger sampling campaign undertaken in Spring 2018, focusing on spatial variation in the AR and its tributaries (Figure 1).^29^ Selection targeted the range of spatial, source-based, and hydrological differences, including DOM- and Fe-rich tributaries (Clearwater R., CW; Horse R., HR; Steepbank R, SB), a transect of surface samples on the east, middle, and west sides of the AR (ARSE, ARSM, and ARSW), and samples collected under high, medium, and base flow conditions following a storm event (HR hiQ and HR, and ARSW hiQ, midQ and lowQ). Satellite coordinates and basic water quality parameters are provided in Supporting Information Table S3.

### Sample Collection and Treatment

All sampling equipment was cleaned using protocols to ensure the absence of contamination, in the metal-free, ultraclean SWAMP lab at the University of Alberta in Edmonton, Canada. Unless otherwise noted, all supplies and reagents were purchased from Thermo Fisher Scientific (Waltham, Massachusetts). Briefly, 125 mL polypropylene (PP) bottles, 20 mL PP syringes, and 125 mL FEP Teflon bottles were cleaned using a 4-step process: (1) soaking in 2% Versa-Clean soap for 24 h and rinsing with ultrapure water (Type II Milli-Q water, 15.1 MΩ cm, Millipore-Sigma, Burlington, Massachusetts); (2) soaking in 5% trace grade HCl for 24 h and rinsing with Type II water; (3) soaking in 10% HNO_3_ for 7 days; and (4) rinsing in triplicate with both Type II and then Type I water (18.2 MΩ cm). All nitric acid was purified in house using sub-boiling double distillation (duoPUR quartz still; Milestone Technologies, Fremont, California). Filters [0.45 μm poly(tetrafluoroethylene)] were cleaned with 2% nitric acid and rinsed with Type I Milli-Q water. The PP tubing used for sample collection was also rinsed with 2% nitric acid and Type I Milli-Q water. All sampling equipment was dried in a dedicated class-100 laminar flow clean air cabinet (NuAire Laboratory Equipment, Plymouth, Minnesota) and double-bagged in Ziploc bags for transport to the field.

Samples were collected using PP tubing and a metal-free closed-loop flow through system, combined with a peristaltic pump (Geopump, Geotech Environmental Equipment, Denver, Colorado). The sampling end consisted of a stainless steel “fish” with a PP sampling nose, through which the sample tubing was routed. The tubing was connected to a stainless steel cable for transporting the sample up to the flow-through sampling system and peristaltic pump, which was housed on a boat. The sample was pumped through the tubing, into the cleaned sample-specific PP transfer bottle through an airtight hole in the lid, out through another hole in the lid to the peristaltic pump, and finally into a flow-through cell to measure water quality parameters (pH, EC, DO, ORP; YSI multimeter, Yellow Springs, Ohio). Sample collection began after enough river water was passed through the transfer bottle to purge the sample tubing and transfer bottle volume 1–2 times. The collected water was transferred and specifically treated for four different analyses: (1) Dissolved TEs: drawn from the transfer bottle into a syringe and filtered directly into an acid-containing PP bottle (final concentration of 2% HNO_3_); (2) AF4-ICPMS: drawn from the transfer bottle into a syringe and filtered directly into an FEP bottle; (3) quasi-total TEs: poured from the transfer bottle directly into an acid-containing PP bottle (final concentration of 2% HNO_3_); and (4) spICP-TOF-MS: capped transfer bottle. The transfer bottle was poured out and rinsed with 1–2 volumes of river water after the collection of each sample to prevent the accumulation of particles in the bottom. Collected samples were stored in coolers with ice packs and transported to the lab for refrigeration at < 5 °C. Analysis using AF4-ICPMS occurred within 2 weeks. Samples for spICP-TOF-MS analysis were frozen for storage and shipping. Dissolved organic carbon concentrations were determined via Total Organic Carbon measurements (Shimadzu TOC-VCPH, Kyoto, Japan). Additional details about the sampling equipment and procedures are provided elsewhere.^7^

### ICP–MS Analysis

All samples were processed, prepared, and analyzed in the SWAMP lab. Sample bottles were opened only when inside class-100 laminar flow clean air cabinets (NuAire Laboratory Equipment). Samples were analyzed inside a class-1000 clean room. Dissolved TE concentrations were measured using quadrupole ICP–MS with high-purity He collision gas (iCAP Qc, Thermo Fisher Scientific). To ensure accurate results and the absence of matrix effects, standard reference materials for TEs in surface waters (SRMs; NIST 1640a and SPS-SW2) were respectively measured at dilution factors of 10-fold and 100-fold and 100-fold and 500-fold after every 12–15 samples (recoveries deemed acceptable when between 80 and 120%). Detection limits were determined as the mean concentration + three times the standard deviation corresponding to the number of counts per second in five blanks of 2% HNO_3_. Instrument running and quality control parameters are provided in the Supporting Information and in previous publications^7,30^ (Tables S3–S6). Samples for quasi-total TE concentrations (i.e., acid-extractable) were first solubilized in pure double-distilled nitric acid using high-pressure and high-temperature microwave-assisted digestion (UltraCLAVE, ATS Scientific). Soil and water SRMs were also digested and analyzed to ensure adequate recovery (NIST 1640a, 2711; SPS-SW2).

### AF4-UV-ICPMS Analysis

Filtered samples were separated along a size continuum using an AF2000 Asymmetric Flow Field-Flow Fractionation system, equipped with an autosampler, UV disinfection, and degasser (Postnova Analytics, Landsberg, Germany). The AF4 was coupled to a UV–visible diode array detector (Agilent 1260 Infinity Series G4212, Agilent Technologies, California, USA) and a quadrupole ICP–MS with He collision gas (Thermo-Fisher iCAP Qc). The operating conditions, quality control measures, and flow program are described briefly in Table S6 and in detail elsewhere^7,30^ (Cuss et al., 2017, 2020b).

### spICP-TOFMS Analysis

spICP-TOFMS was collected at the University of Vienna. All AR and tributary samples were analyzed using an icpTOF 2R (TOFWERK AG, Thun, Switzerland). The ICP-TOF is capable of measuring the majority of the atomic mass range (7–250 *m*/*z*^+^), with a mass-resolving power of 6000 full width at half-maximum and a TOF extraction frequency of 46 kHz. This ICP-TOF utilizes a notch filter, which attenuates up to four chosen masses, in this case, ^40^Ar^+^, ^16^O_2_^+^, ^35^Cl^+^, and ^1^H^+^, which are unimportant for the analysis yet comprise a significant portion of the ion beam.

To improve the signal-to-noise ratio of ^56^Fe^+^ and ^28^Si^+^, a 7% H_2_/He mixture was used as a collision gas with flow parameters optimized before analysis for maximum sensitivity (Supporting Information Table S7). Calibration of the instrument was performed daily using Tune solution B (ThermoFisher) containing one μg/L Li, Co, Ce, In, Ba, Bi, U. Recalibration was performed regularly to account for instrumental drift that might occur throughout a run. Dissolved calibration solutions were prepared from ESI stock solution of dissolved metals (Al, Si, Fe, Cu, Ti, Zn, Pb, Cd, Cu, Nd, and Ni), analyzed prior to each sample run, with a continuing check verification standard every 10 samples to account for any drift in instrument sensitivity. Additional and typical operating parameters are listed in Supporting Information. A 100 nm gold nanoparticle dispersion (BBI solutions) was used as a known mass standard for obtaining transport efficiency. Despite a minimum dwell time of 46 μs for the icpTOF 2r, a 3 ms dwell time was used due to limitations on data transfer from the data acquisition system to the laboratory computer. To avoid particle coincidence, a 1000 X dilution of the samples was required.

The raw mass spectrum data were initially processed using TofWare (TOFWERK AG, Thun, Switzerland), which allowed for peak integration after initial subtraction of the spectral baseline. The resulting data were then processed via a custom Python script, which performed calibration and spectral correction of the data. The script also then processed the single particle data according to the previously established methodology.^31−33^ The subsequent data output resulted in a compiled list of particle events with time and their associated masses, which can then be converted into mass and size according to the single particle theory.

### X-ray Diffraction and Scanning Electron Microscopy Analysis

The dominant mineral phases in the sediments were determined using random powder X-ray diffraction (XRD) (RIGAKU Ultima IV) with a cobalt tube at 38 kV as a source and 38 mA and D/Tex Ultra with an Fe Filter (K-beta) detector at the University of Alberta. Fine powdered sediment samples were loaded onto silicon zero background plates using ethanol and scanned using Co Kα X-ray from 5 to 90 2θ with a continuous scan mode, 0.0200° sampling width (step size) at 2.00° per minute scan speed. Data interpretation was performed using DIFFRAC.EVA software with the 2020 ICDD PDF 4+ and PDF 4+/Organics databases.

To determine the micromorphology and elemental composition of the suspended sediments in the AR, 0.45 μm disc filters used in the field for dissolved TEs were cut open to acquire the sample. A precision lathe was customized to cut the filter discs to the exact depth needed to completely recover the filter membrane and minimize particle loss and the risk of sample contamination. The details of the method were provided previously.^34^ Prior to cutting, all filters were vacuum-dried to remove water and ensure that the particles remained attached to the membrane. A high spatial resolution (∼10 nm) Zeiss Sigma 300 VP-FESEM scanning electron microscope equipped with a backscattered electron detector and Bruker energy-dispersive X-ray spectrometer with dual silicon drift detectors each with an area of 60 mm^2^ and a resolution of 123 eV was used at the University of Alberta. Filter membranes containing the sediment were mounted on an Al stub using a double-sided carbon tape, coated with gold to prevent the charging on the sample surface due to the electron beam, and examined under SEM.

## Results and Discussion

### New Insights Using the AR as a Case Study

Complexity in the nanogeochemistry of natural riverine environments is driven by local watershed geology, sediment erosion, chemical weathering, in-stream particle formation/transformation, anthropogenic influences, and organic carbon biogeochemistry. Certain chemical parameters such as the quantity and quality of dissolved organic carbon (DOC) can have an outsized influence on the formation, mineralogy, and structure of NNPs, for example, impacting the formation and stability of iron oxides at oxic–anoxic interfaces.^36^ A previous work indicates that tributaries of the AR contain up to three times as much DOC compared to AR mainstem, potentially driving the measured differences in NNP size, number, and mass distributions.^7,10^ The geochemical cycling of metal oxides and watershed contributions of silicate minerals are also relevant to the particulate transport of potentially toxic elements in the AR, such as Pb (Table 1).^34^ The nanogeochemical environment of the AR and its tributaries were thus considered a good candidate for investigation using AF4-ICP-MS and spICP-TOFMS in tandem (Figure 1, Supporting Information Figures S1 and S2). Given this geochemical context, three broad topics were selected to demonstrate its potential: (1) the complexity of particle mineralogy, as evidenced by major elements in particles approximately <5 μm; (2) the distribution of a common TE of concern (Pb) among particles with different sizes and compositions, and (3) the potential for particle-by-particle isotopic and forensic analyses.

**Table 1:** Number of Particle Events Detected by spICP-TOF-MS for Different Combinations of Si, Al, Fe^a^

		Si	Al	Fe	Si, Al	Si, Fe	Fe, Al	Si, Al, Fe	Pb only
HR hiQ	total	336 ± 24	316 ± 9	672 ± 26	155 ± 15	146 ± 12	235 ± 9	573 ± 23	
	w/Pb	**	1 ± 2	11 ± 4	2 ± 1	2 ± 1	4 ± 1	17 ± 4	5 ± 4
HR	total	95 ± 3	185 ± 12	508 ± 31	34 ± 4	29 ± 7	141 ± 16	170 ± 19	
	w/Pb	**	2 ± 1	1 ± 1	1 ± 1	**	1 ± 1	2 ± 3	2 ± 1
CW	total	95 ± 3	179 ± 22	907 ± 24	43 ± 8	54 ± 6	253 ± 10	249 ± 17	
	w/Pb	**	**	1 ± 1	**	**	2 ± 1	4 ± 1	2 ± 1
ARSW lowQ	total	150 ± 9	221 ± 23	1122 ± 34	74 ± 4	122 ± 11	328 ± 3	473 ± 13	
	w/Pb	**	1 ± 1	6 ± 1	1 ± 1	2 ± 1	2 ± 1	10 ± 7	5 ± 1
ARSW midQ	total	529 ± 31	286 ± 15	689 ± 21	207 ± 9	233 ± 3	167 ± 11	555 ± 20	
	w/Pb	4 ± 4	3 ± 3	38 ± 2	1 ± 1	8 ± 3	4 ± 0	28 ± 2	43 ± 5
ARSW hiQ	total	540 ± 56	300 ± 36	744 ± 20	201 ± 10	237 ± 12	186 ± 32	573 ± 361	
	w/Pb	2 ± 2	2 ± 1	33 ± 7	2 ± 2	10 ± 5	6 ± 2	26 ± 6	38 ± 15
SB	total	35 ± 2	28 ± 4	223 ± 3	3 ± 0	6 ± 2	42 ± 3	41 ± 1	
	w/Pb	**	**	**	**	**	**	**	**

a Tabulated events are categorized by both total particle events detected and those specifically exhibiting a concurrent Pb detection event. Values represent the average and standard deviation across *n* = 3 replicates. ** Pb not detected.

### Major Element Distributions Show Diverse Mineral Populations

Riverine particles can originate from the surrounding watershed or may be formed in situ through (bio)geochemical processes. The former depends on hydrologic conditions, whereas the latter reflects water chemistry. Particles introduced from the weathering of crustal material are generally dominated by aluminosilicate minerals. Common silicate minerals, especially clays, have Si/Al mole ratios in the range of 1–2 that are specific to their mineral structure and the degree of Al and Si substitution in the crystal lattice. The compositions of aluminosilicates thus serve as indicators of geochemical origin (physical and or chemical weathering) and provide insights into watershed-scale transport and geochemical processes.

**Figure 2:**
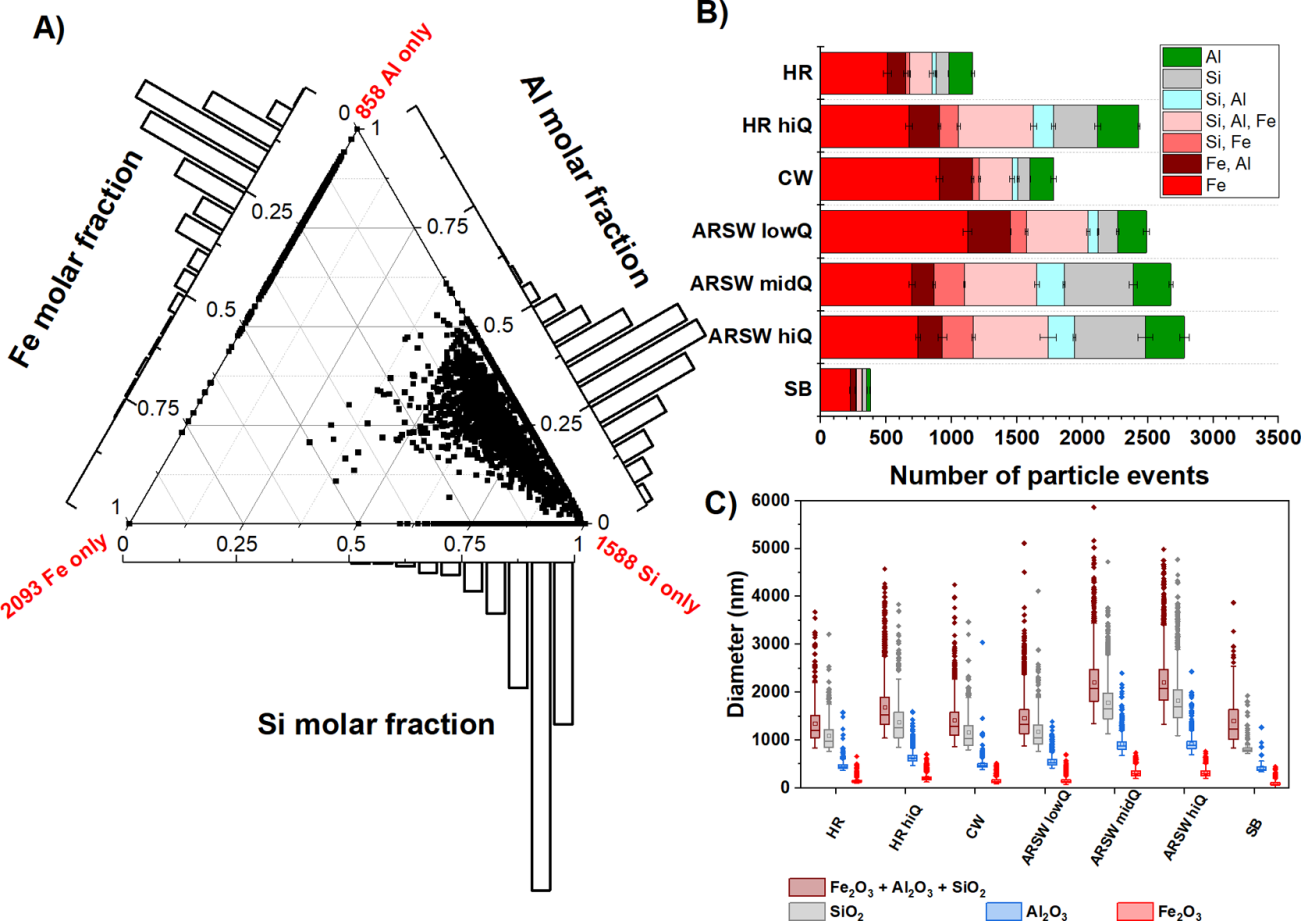
Results from spICP-TOF-MS analysis of silicate and metal oxide particulate signals from the ARSW and its tributaries. (A) Ternary plot detailing molar distribution of Fe, Al, and Si on a single particle basis, generated using triplicate analysis of sample ARSW midQ. Each point represents an individually detected particle event, while histograms on the axis detail the molar distribution of Al–Si, Al–Fe, and Si–Fe. The number of single element detection events are listed on the vertices of the plot. (B) Average particle events of Fe, Si, and Al and combinations thereof for selected sample sites. (C) Computed sizes for Al, Si, and Fe particle events assuming oxide formulas and spherical geometry.

Applied to unfiltered samples, the multi-element analysis capabilities of spICP-TOFMS provided quantification of particles containing detectable combinations of Al, Si, and Fe (Figure 2) and can be further categorized into particles containing a detectable Pb mass (Table 1). Determination of K, Ca, Mg, and Na was impaired by isobaric interferences from Ar molecular ions, insufficient sensitivity, and low abundance. An example ternary plot (Figure 2A) shows the Fe/Al/Si particle composition for the AR sample ARSW lowQ (ARSW Surface West at low flow (Q), additional samples are shown in Supporting Information Figure S3). The ternary plots demonstrate several outstanding features of spICP-TOFMS. For example, the measurement of elemental compositions in hundreds to thousands of particles requires only 1–5 min of analysis using a few mL of sample. This analysis facilitates thorough characterization and comparison of composition at the scale of individual particles, revealing both the diversity of aluminosilicate particle compositions and the pervasiveness of Fe in the NNP of this system. The widely ranging Al/Fe/Si composition (Figure 2A, B) demonstrates a highly diverse mixture of minerals, some with compositions intermediate from the likely pure mineral components. Likely explanations for these observations are the presence of surface coatings of Fe and/or Al, or heteroaggregates of different mineral particles. It must be noted that while all seven potential combinations of elements were observed with varying degrees in each sample, “single-element” (e.g., Fe-only) and “bi-element” (e.g., Fe/Al) minerals may contain an additional element that has insufficient mass to be detected. The ability to detect a minor element in a particle is a combination of both its content in the particle and the elemental mass detection limit (Table S1). Additionally, the power law relationship between size and particle number concentration and inability of ICP–MS to transport and ablate particles above about 5 μm limits us to observing particles primarily in the sub-micron size range (e.g., Figure 2C). Despite these limitations, the spICP-TOFMS analysis of riverine nanoparticles and colloids provides deeper insights into their origins and dynamics.

Elemental analysis alone does not unambiguously identify specific mineralogy, especially when not all elements (Ca, K, Na, Mg, and O) are measured; however, a number of insights are possible, which cannot be easily achieved using traditional bulk techniques like XRD (Supporting Information Figure S4) or SEM–EDX (Supporting Information Figure S5). For example, spICP-TOFMS analysis indicated that these samples were dominated by particles containing only detectable iron (Figure 2A,B; Table 1). The presence and formation of metal oxides have a significant effect on TE speciation and could thus lead to major differences among these rivers. Except for ARSW lowQ, tributaries contained higher proportions of Fe-dominated particles (Figure 2B-red shaded bars) and a lower proportion of Si-containing particles. Given that the tributaries generally had higher concentrations of DOC (Table S2) compared to the main stem, this could indicate the influence of DOC-mediated iron redox cycling in the tributaries (CR, SB, and HR), compared to hydrologically driven sediment transport in the main stem of the AR.^7^ The majority of tri-element particles had Al/Si ratios < 0.5, and XRD analysis showed broad peaks consistent with 1:1 and 2:1 clay minerals (Figure S4). These particles generally contained Fe mole fractions <0.25, and the Fe-containing, bi-element particles likewise contained small proportions of Fe (<0.5 mol fraction). This suggests its presence as coatings on Fe/Si particles or as a minor substitution in Fe/Al particles. In contrast, SEM–EDX analysis did not detect any single- or bi-element particles (Supporting Information Figure S5). For the Al-only and Si-only particles, this may result from a combination of small numbers of particles examined by SEM–EDX, and their low abundance (<20% of the total particles). It is more difficult to explain the absence of apparent Fe-only particles in SEM–EDX, as they were the most frequent particle composition indicated by analysis using spICP-TOFMS. Particles may be subject to heteroaggregation during preparation for SEM–EDX, resulting in artefactual particles containing all three elements, as suggested by the greater Fe content of three-element particles measured using SEM–EDX (Supporting Information Figure S6). The absence of Al–Si– and Fe– only particles detected by EDX could also be attributed to its inability to resolve single particles, and the corresponding combination of element signals from neighboring particles, as well as relatively high detection limits of EDX (∼0.1 wt %).

A very significant output of spICP-TOFMS is the estimation of particle sizes, their distributions, and the corresponding distributions of elements therein. These sizes are calculated according to spICP-MS theory that has been described elsewhere.^31,32^ Briefly, the Al, Fe, and Si intensities are converted to mass based on a mass flux curve that is produced with a set of dissolved standards and a measured transport efficiency. These masses can then be used to calculate a diameter using an estimated mass fraction from the known oxide formula (SiO_2_, Fe_2_O_3_, and Al_2_O_3_). The equivalent spherical diameters are then computed assuming a spherical geometry and the density of the chosen oxides (Figure 2C). This provides a rough size estimate, as well as relative sizes for comparison of sampling locations. The measured Fe-only particles were relatively small (100–700 nm) compared to the Al- and Si-containing particles (mostly >1 μm). This may also explain why Fe-only particles were not apparent using SEM–EDX, as they may have been more difficult to detect among the larger particles. The distribution of particles in the AR and HR (Horse River) also shifted somewhat toward larger sizes with increasing flow rates, consistent with an erosional origin for the larger aluminosilicates and a biogeochemical origin for the smaller iron-dominated particles.

### Lead Transport is Facilitated by Natural Nanoparticles Containing Iron and Manganese

**Figure 3:**
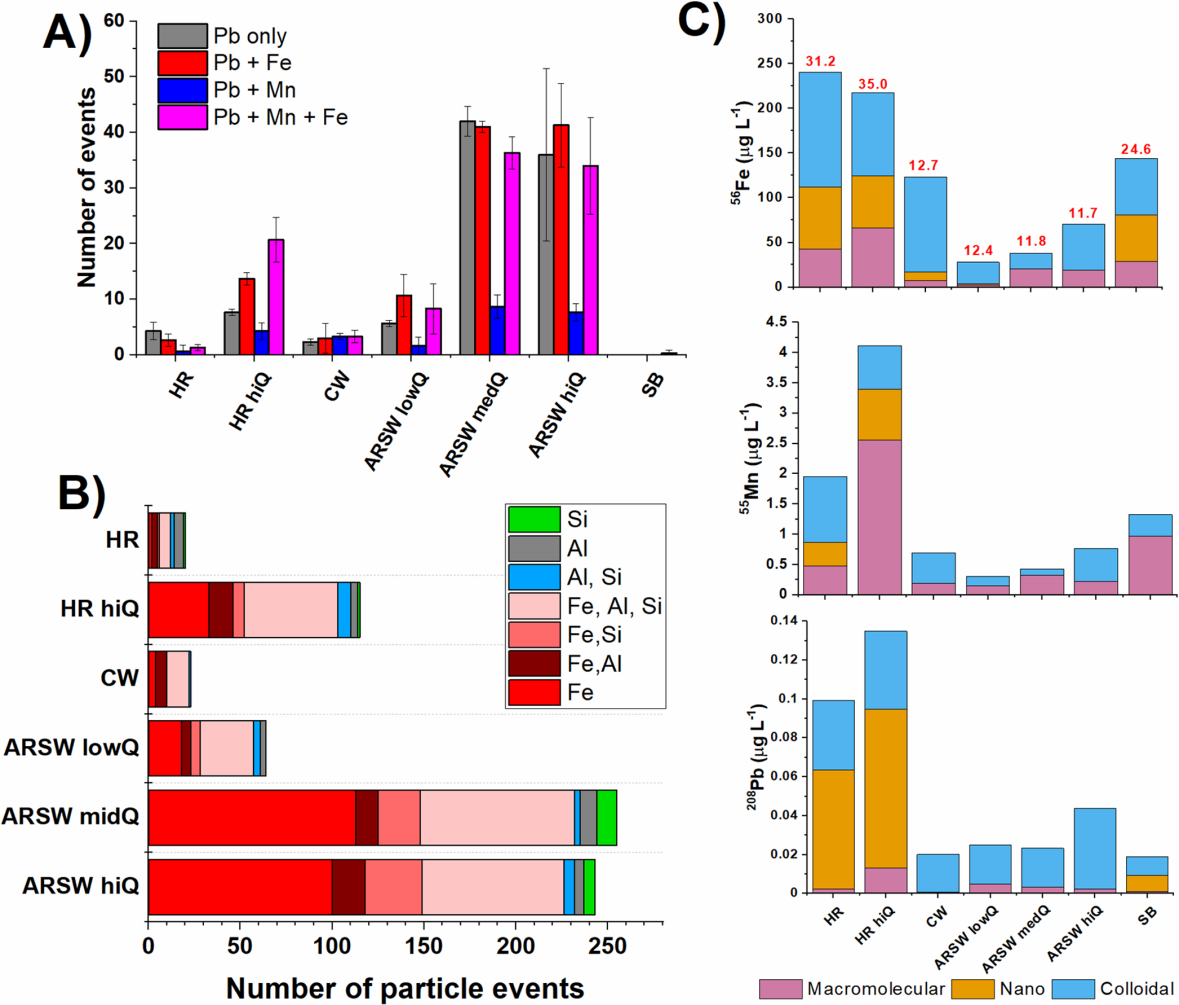
Distribution of Pb-containing particles in the ARSW and tributaries. (A) Number of spICP-TOF-MS particle detection events containing Pb, Fe, and Mn. (B) Total number of lead-containing particle events sorted by co-occurrence with Fe, Si, and Al and combination thereof. (C) Concentrations of Fe, Mn, and Pb in the macromolecular, nano, and colloidal fractions as determined by AF4-ICPMS. Dissolved organic carbon concentrations (mg L^–1^) are shown in red above the Fe bar chart.

The spICP-TOFMS analysis suggested that Pb transport is perhaps facilitated by its association with Fe- and Mn-containing particles (Figure 3A,B). The impact of flow rate was readily apparent in the greater number and size of particles observed in higher flow regimes (ARSW medQ and hiQ, HR hiQ). The importance of Fe was also apparent for Pb transport in aluminosilicate particles, wherein Pb was predominately associated only with those particles that also contained Fe (Table 1, Figure 3B-red shaded bars). While the possibility of additional Al–Pb only particles cannot be ruled out, results indicate that Fe plays a primary role in the nanophase transport of Pb in these rivers.

Sizes of detected Fe particles, Fe–Pb particles, and Fe–Mn–Pb particles were calculated using the detected Fe mass, an assumed spherical shape and hematite minerology (Fe_2_O_3_), and an assumption that insignificant mass is contributed by Pb and Mn. The association of Pb and Mn with only larger particles may be the consequence of detection limits; that is, exceptionally small amounts of Pb and Mn may not be measurable in the smaller Fe-containing particles. Figure 3A also demonstrates relatively low particle number concentrations in the tributaries, suggesting biogeochemical sources. High DOC in the tributaries (Supporting Information Table S2) may act to stabilize smaller particles, preventing aggregation and particle growth,^26,30^ as suggested by conservation of the concentration and colloidal distribution of dissolved Fe from tributaries during mixing with the mainstem over 60–100 km.^35,36^ Larger particle sizes in the AR suggest another source such as erosion, or the aggregation of upstream sources.

In this study, AF4-ICPMS provided quantification of Fe, Mn, and Pb in inorganic phases ca. 3–450 nm in size and macromolecules (DOC) spanning 0.3–5 kDa in size, or ca. 1–3 nm. The high concentrations of particles observed illustrates how defining the <0.45 μm fraction as “dissolved” misrepresents the nature of TEs in this size range (Figure 3C). The bulk of Pb in the <0.45 μm fraction was transported on metal oxide nanoparticles (3–100 nm) and small colloids (100–450 nm), with only a small amount present in the macromolecular (organic) fraction. In contrast, Fe and Mn were present in all fractions, with higher percentages present in inorganic colloids for the AR mainstem. AF4-ICP-MS analysis elucidates a complexity of phases within the <0.45 μm fraction that is both element specific and related to sample location. The concentration of Fe-containing particles was higher in the AR when measured for larger particles using spICP-TOFMS, but the concentration of Fe-containing particles was higher in the tributaries when measured using AF4-ICPMS (Figure 3B,C). The different source-based distributions of Fe within these smaller (AF4-ICPMS) and larger (spICP-TOFMS) size fractions indicate the need to use both techniques to obtain a complete picture of the nanogeochemical environment.

### Multi-Element, Single Particle Analysis Offers New Avenues of Research in Isotope Geochemistry and Environmental Forensics

**Figure 4:**
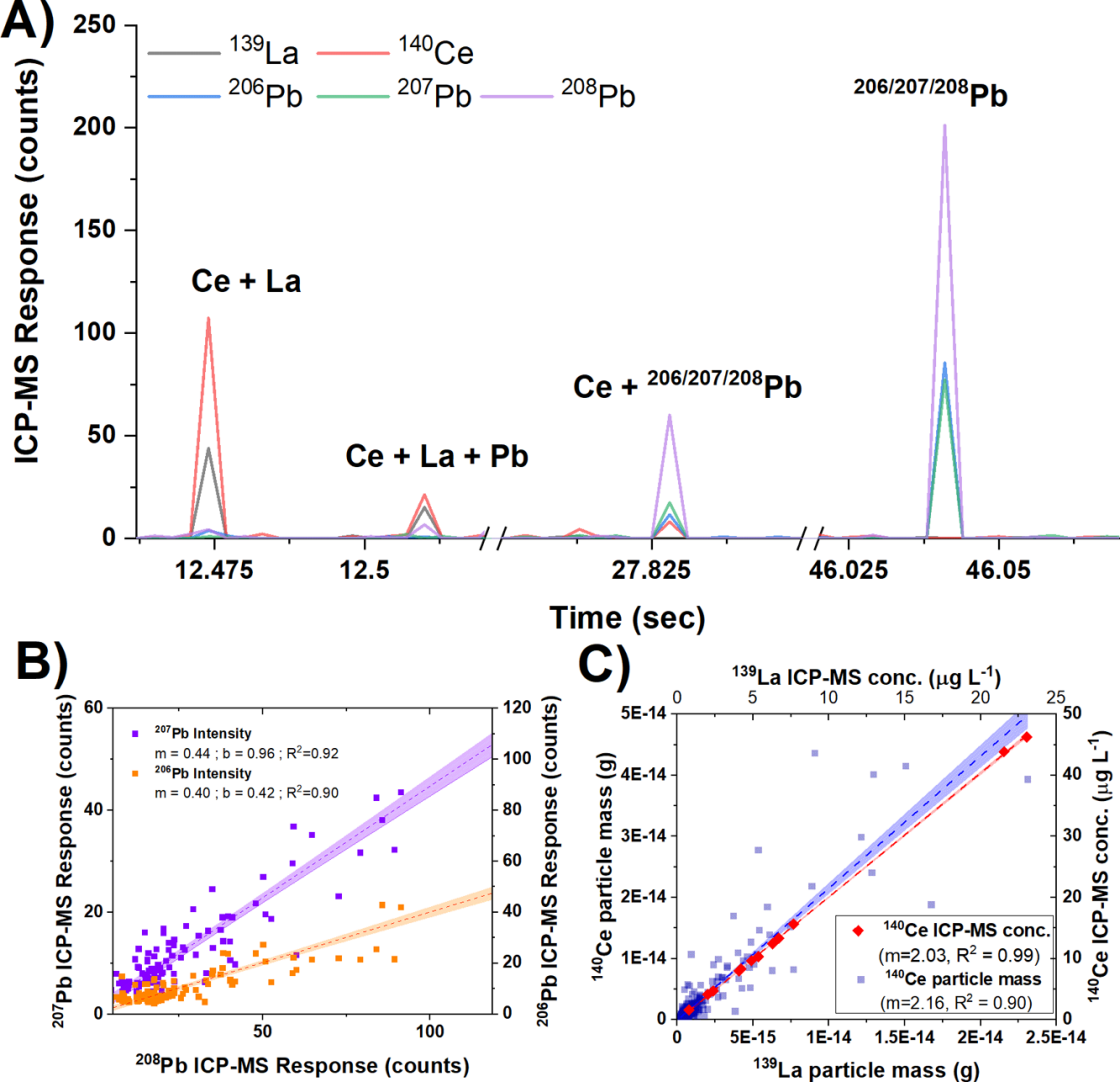
Examples of spICP-TOFMS applications to TE geochemistry. (A) Selected peaks in ARSW midQ showing particles with elemental ratios of Ce and La and isotopic ratios of Pb that are consistent with natural abundance. (B) Isotopic ratios of Pb for all detected particles with linear regression approximating natural isotopic abundance. (C) Ce and La ratios for all detected particles plotted against total ICP–MS concentrations from each site.

An important output of spICP-TOFMS analysis is the identification of elemental composition of individual particles and their classification into single- and multi-elemental particles (Figure S7). An as yet unexploited application is the measurement of the isotopic composition of elements within individual particles. This is only possible if sufficient masses of each isotope are present. Selected particle detections for site ARSW midQ illustrate the co-occurrence of Ce and La and the distribution of Pb isotopes (Figure 4A). While these capabilities have been used elsewhere in attempts to distinguish naturally occurring and ENP populations,^23,24^ the present data demonstrate the potential of this technique to address broad nanogeochemical questions in natural systems. The mean Ce/La ratios measured using spICP-TOFMS (Ce/La = 2.16) also compare well to those obtained from traditional ICP–MS analysis of the <0.45 μm fraction (Ce/La = 2.03). The Pb isotopic ratios from particle events (slopes in Figure 4B) were within the range of measured isotopic ratios for 206/208 Pb and 207/208 Pb in surface waters, respectively.^37^ The observed scatter in these ratios suggest a level of considerable variation in particle composition that is not observable by bulk ICP–MS, raising new and exciting questions for nanogeoscience (Figure 4B,C). These data clearly demonstrate the ability of spICP-TOFMS to measure elemental and isotopic particle compositions on a single particle basis, providing exciting new possible applications in environmental forensics and source tracking.

## Conclusions

This work demonstrates that particle-by-particle analysis provides new insights into the nanogeochemical environment in natural waters and a range of potential applications including environmental forensics, risk assessment, and the study of fundamental geochemical processes. The single-element particles measured in tributaries comprised 80% of the particle population but only 60% in the main stem, suggesting different (bio)geochemical origins and hinting at the power of spICP-TOFMS for use in environmental forensics. The highly variable major element composition of observed particles indicated that particle composition was far more diverse than apparent from bulk mineralogical analysis. This variation was also present in the ratios of Ce/La and Pb isotopes. Finally, the relative influence of specific carriers such as Al, Fe, and Mn on TE (Pb) transport can be directly quantified.

Both the importance of nanoparticles in natural geochemical cycles and the need to establish their current abundance and composition in order to quantify the impacts of anthropogenically induced changes support the need for these improved analytical capabilities. The combination of spICP-TOFMS and AF4-ICPMS enables the multi-element analysis of an expansive range of particle sizes and composition. Single particle ICP-TOFMS in particular facilitates the study of elemental and isotopic ratios within individual particles, a capability typically reserved for electron microscopy and X-ray spectroscopy, which are severely challenged with respect to obtaining a representative sample. Although spICP-TOF-MS represents a major advancement that will prove useful to earth science, the quantification of elemental and isotopic signatures is currently subject to element-specific detection limits, wherein low masses of an element within a particle may not be detected. This challenge could be overcome through appropriate data fusion with more sensitive but throughput-limited techniques such as ICP–MS or TEM. Despite these hurdles, the high-throughput of spICP-TOFMS and appreciable sensitivity for the majority of metallic elements provides data commensurate with bulk measurements and will likely be improved as the technique gains widespread accessibility and use.

## Supporting Information Available

Analytical operating conditions and sampling information, additional FFF–ICP–MS and FFF–UV–vis fractograms, ternary diagrams for each of the described sampling sites, XRD and SEM images from selected sites, dissolved organic carbon, total metals, and filtered metals concentrations, operating conditions for the instrumentation, and additional figures of merit for spICP-TOF-MS (e.g., equivalent size detection limits) (PDF)

## Abbreviations

AF4: asymmetric flow field flow fractionation
AR: Athabasca River
DOC: dissolved organic carbon
ENP: engineered nanoparticle
NNP: natural nanoparticle
SEM-EDX: scanning electron microscopy-energy dispersive X-ray spectroscopy
spICP-TOFMS: single particle inductively coupled plasma-time-of-flight-mass spectrometry
TE: trace elements
TEM: transmission electron microscopy
XRD: X-ray diffraction
