# Supplementary material for: Exploring Nanogeochemical Environments: New Insights from Single Particle ICP-TOFMS and AF4-ICPMS
Source: ACS Earth Space Chem. 2022 Apr 4;6(4):943–52. doi: 10.1021/acsearthspacechem.1c00350 (PMC9037182; doi:10.1021/acsearthspacechem.1c00350)
Supplement: Supplementary file 1 — sp1c00350_si_001.pdf [file sp1c00350_si_001.pdf]

Exploring nanogeochemical environments: new insights from single particle ICP-TOFMS and AF4-ICPMS

Manuel D. Montaño,[1,*] Chad W. Cuss,[2,3] Haley M. Holliday,[4] Muhammad B. Javed,[2] William Shotyk,[2] Kathryn L. Sobocinski,[1] Thilo Hofmann,[5] Frank von der Kammer,[5] James F. Ranville[6]

[1]Department of Environmental Science, Western Washington University, Bellingham, WA, U.S.A. 98225
[2]SWAMP Laboratory, Department of Renewable Resources, University of Alberta, Edmonton, Alberta, Canada, T6G 2H1
[3]School of Science and the Environment, Memorial University of Newfoundland Grenfell Campus, Corner Brook, Newfoundland, Canada, A2H 5G4
[4]Department of Chemistry, Western Washington University, Bellingham, WA, U.S.A. 98225
[5]Centre for Microbiology and Environmental Systems, Department of Environmental Sciences, University of Vienna, Vienna, Austria 1090
[6]Department of Chemistry, Colorado School of Mines, Golden, CO, U.S.A. 80401

Figure S1. FFF fractograms for Fe, Mn, Al, Pb
Figure S2. FFF fractograms for DOC
Figure S3. Ternary diagram of Fe, Al, Si molar ratios based from SEM-EDX data
Figure S4. XRD for selected samples
Figure S5. SEM for selected samples
Figure S6. Fe, Al, Si Ternaries for all sites
Figure S7. Proportion of multi- and single-element particles identified in ARSW midQ
Table S1. Calculated minimum size detection limits by element/isotope
Table S2. Dissolved organic carbon concentrations
Table S3. Sampling details for selected sites
Table S4. Total ICP-MS data
Table S5. Filtered ICP-MS Data
Table S6. Operating conditions for AF4 and ICP-MS (total and dissolved)
Table S7. Operating conditions for spICP-TOFMS
Figure S8. Raw counts vs time single particle data
Figure S9. Calculated hematite sizes for particles containing iron and manganese

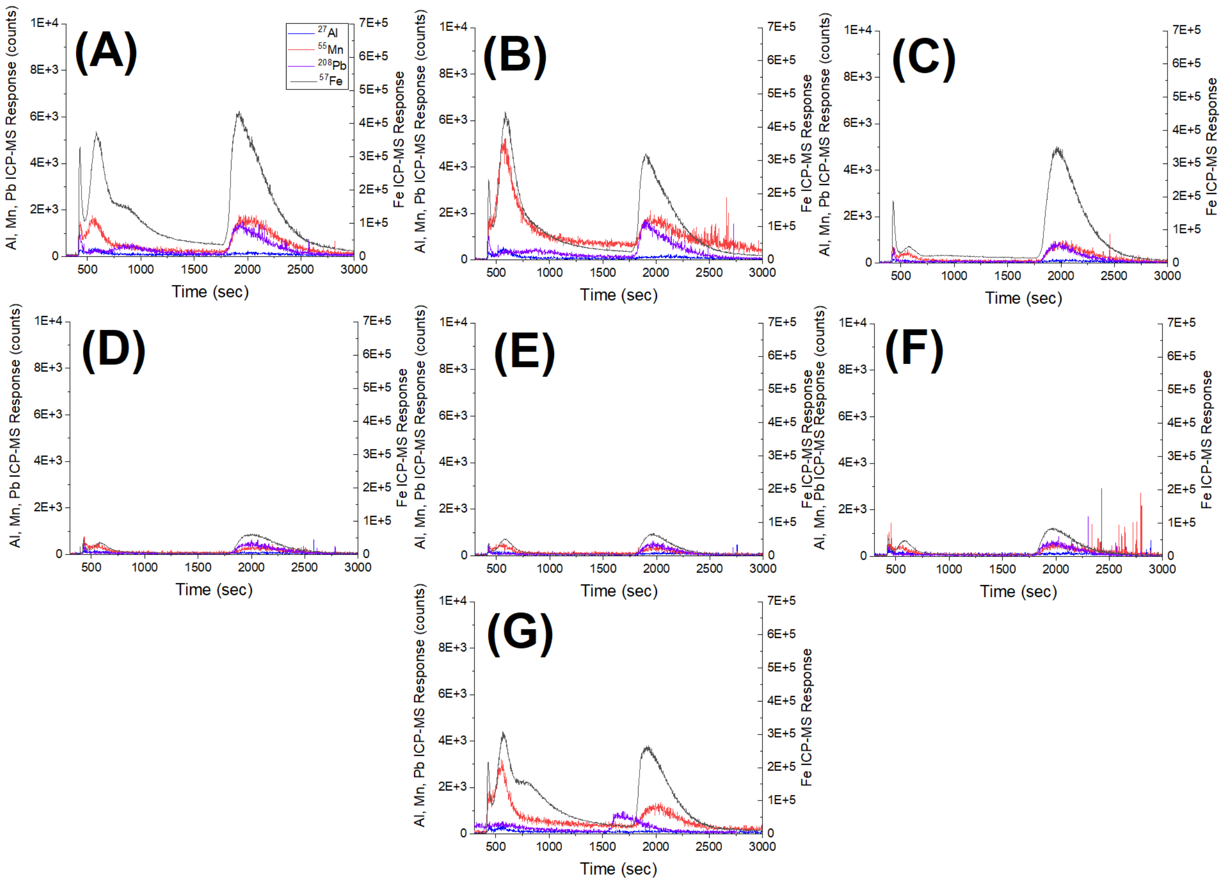

Figure S1. AF4-ICP-MS Fractograms for selected sites. (**—,** right y-axis) indicates ^{57}Fe signal. Elements on the left y-axis are: (—) ^{27}Al signal, (—) ^{55}Mn signal, (—) ^{208}Pb signal. Samples were pre-filtered by 0.45 µm prior to analysis. Sites are listed as follows (A) Horse River, (B) Horse river high flow, (C) Clearwater River, (D) Athabasca River Middle Surface low flow, (E) Athabasca River Middle Surface middling flow, (F) Athabasca River Middle Surface high flow, (G) Steepbank River.

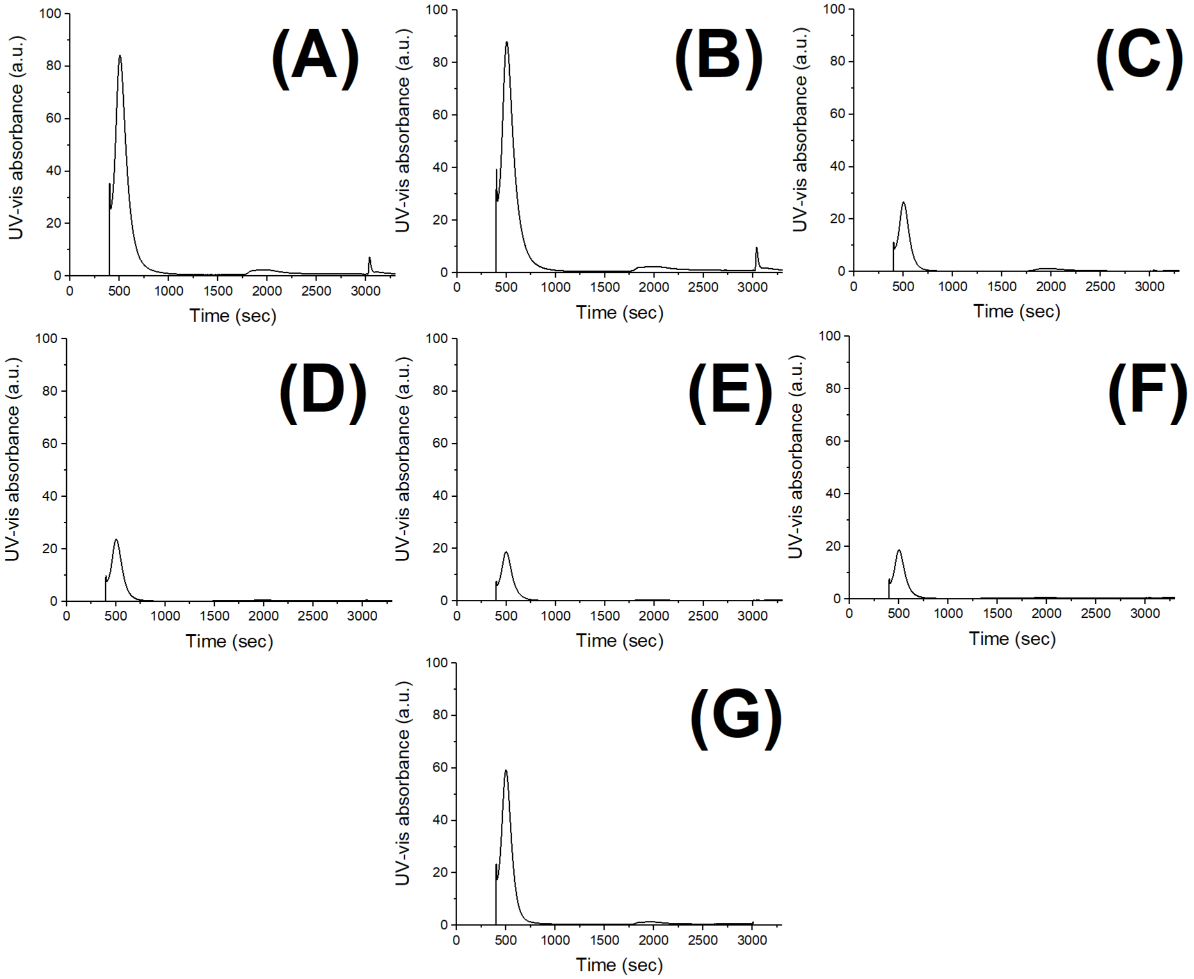

Figure S2. AF4-UV-vis Fractograms for selected sites. Samples were pre-filtered by 0.45 µm prior to analysis. Sites are listed as follows (A) Horse River, (B) Horse river high flow, (C) Clearwater River, (D) Athabasca River Middle Surface low flow, (E) Athabasca River Middle Surface middling flow, (F) Athabasca River Middle Surface high flow, (G) Steepbank River

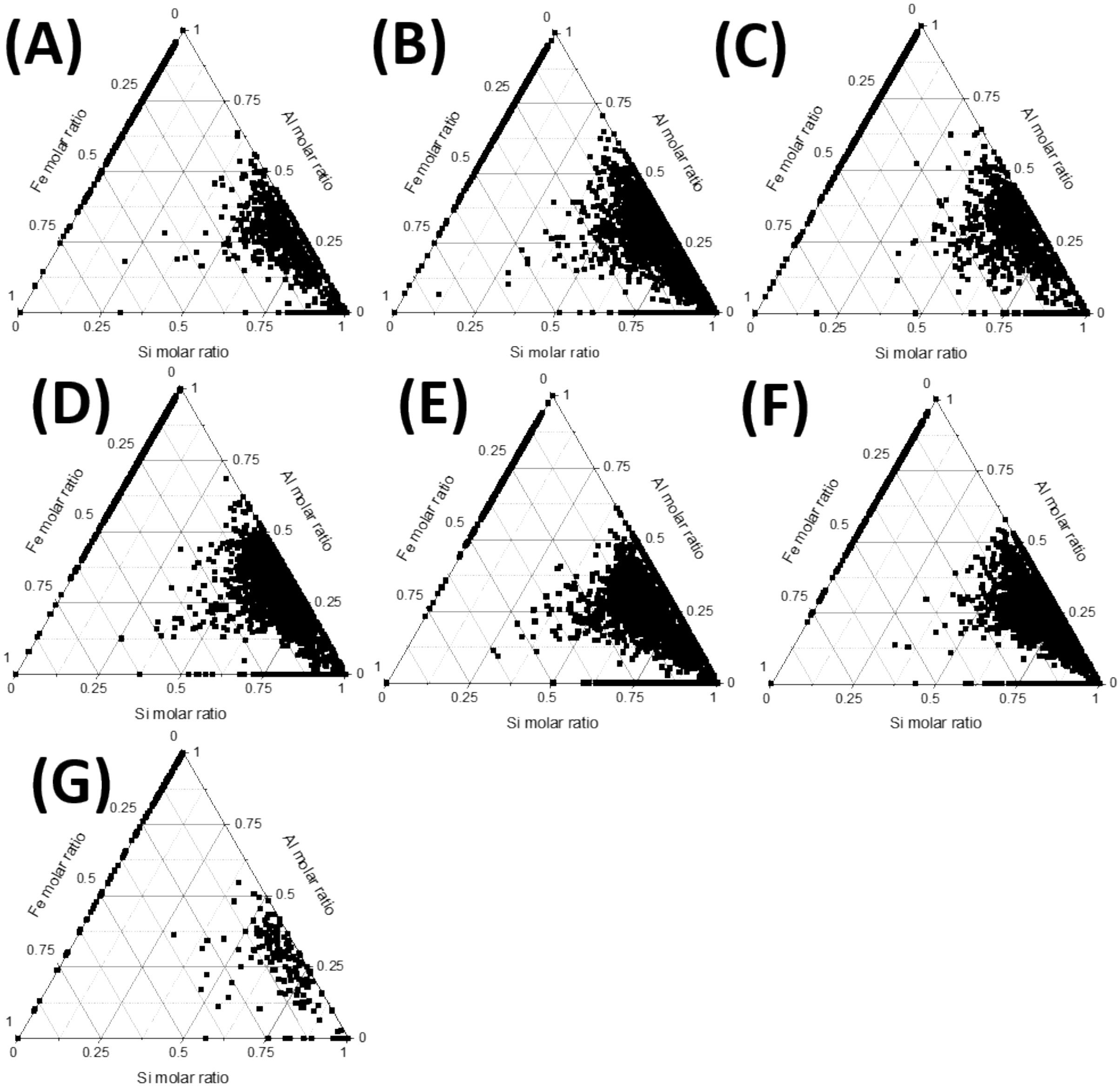

Figure S3. Aluminum, silicon, iron molar ratio ternary diagrams for each selected sample. (A) Horse river, (B) Horse river hiQ, (C) Clearwater river, (D) ARSW lowQ, (E) ARSW midQ, (F) ARSW hiQ, (G) Steepbank river

Table S1. Calculated minimum size detection limits for various isotopes used in this study. All sizes assume spherical geometry, unity mass fraction, and an assumed density of the pure element.

**Element/Isotope**	D_{min}
**27Al**	81
**28Si**	311
**47Ti**	90
**48Ti**	42
**51V**	29
**55Mn**	19
**56Fe**	20
**57Fe**	212
**60Ni**	22
**63Cu**	27
**64Zn**	37
**68Zn**	57
**75As**	53
**112Cd**	34
**121Sb**	16
**123Sb**	17
**138Ba**	13
**139La**	3
**140Ce**	10
**184W**	6
**197Au**	6
**206Pb**	11
**207Pb**	11
**208Pb**	8
**232Th**	6

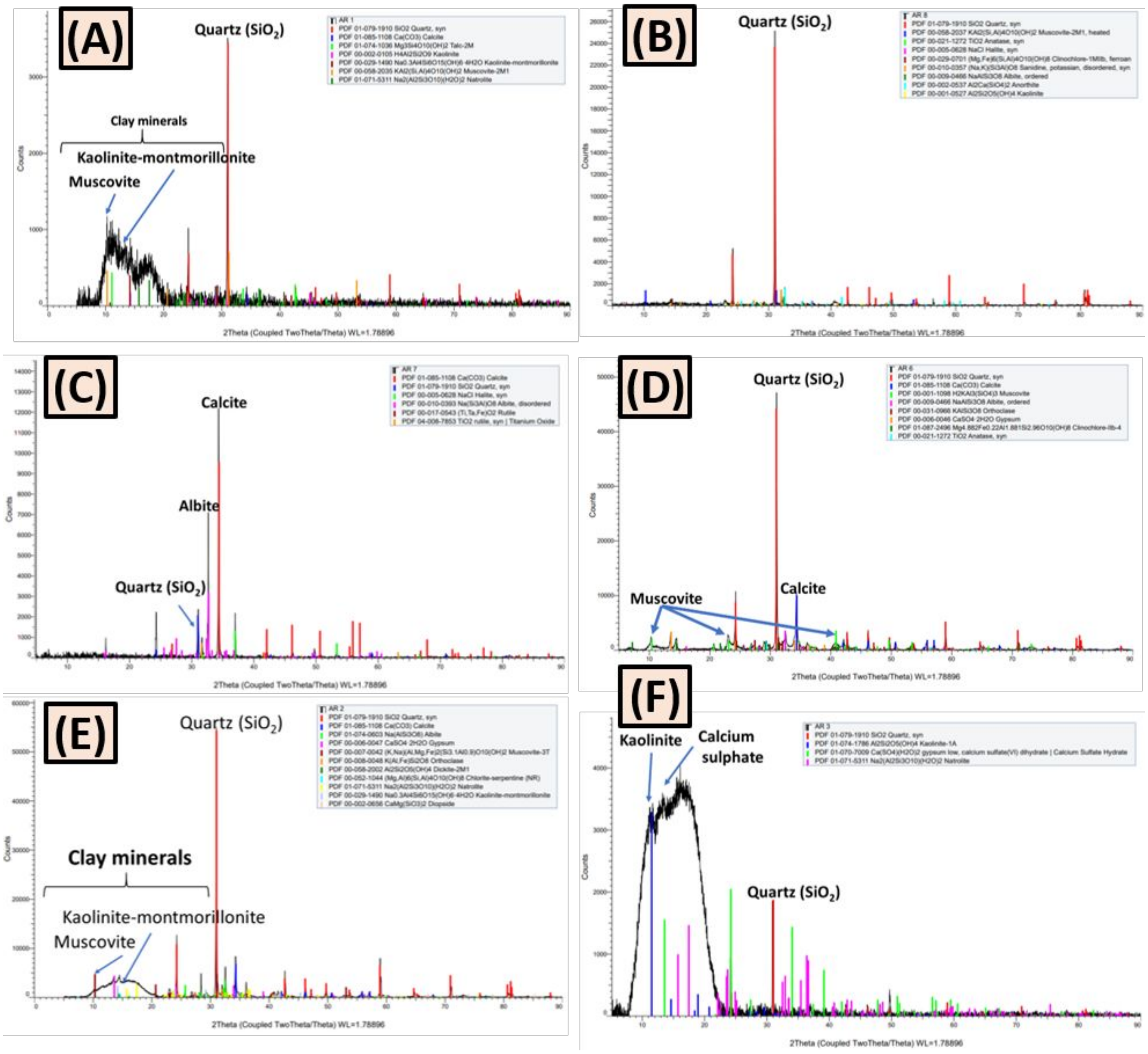

Figure S4. Powder XRD of selected sites as described in materials and methods. Sites are listed as follows (A) Horse River, (B) Horse river high flow, (C) Athabasca River Middle Surface low flow, (D) Athabasca River Middle Surface middling flow, (E) Athabasca River Middle Surface high flow, (F) Steepbank River

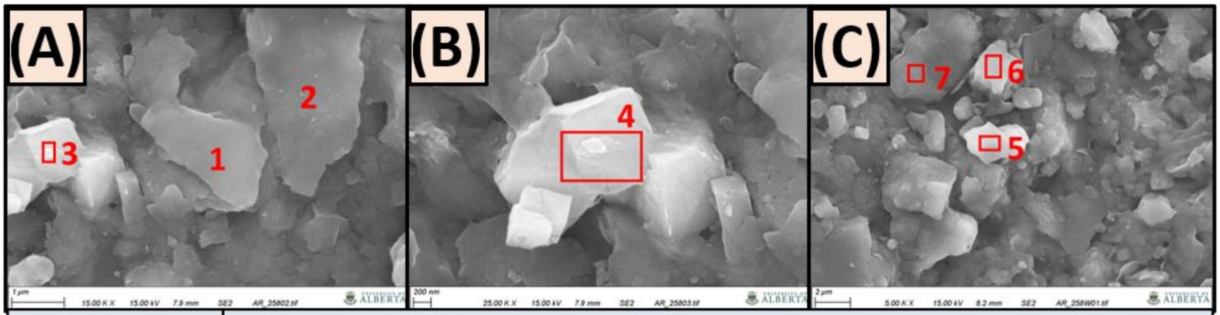

Spectrum	Elemental Composition – EDX (%)								
	C	O	Na	Mg	Al	Si	K	Ca	Fe
1-a	9.3	48.7	0.2	1.4	11.9	19.3	5.2	0.8	3.2
2-a	9.7	47.5	0.2	1.4	10.7	22.4	3.1	1.3	3.7
3-a	9.8	46.9	2.9	0.5	2.6	5.5	2.1		21.0
4-b	9.1	46.1	2.8	0.5	2.9	6.0	2.0	0.6	21.5
5-c	8.3	51.9			2.1	37.7			
6-c	5.6	53.0		0.4	15.9	22.0	0.4	0.5	2.4
7-c	5.6	49.4		1.1	13.6	24.6	0.8	1.0	4.0

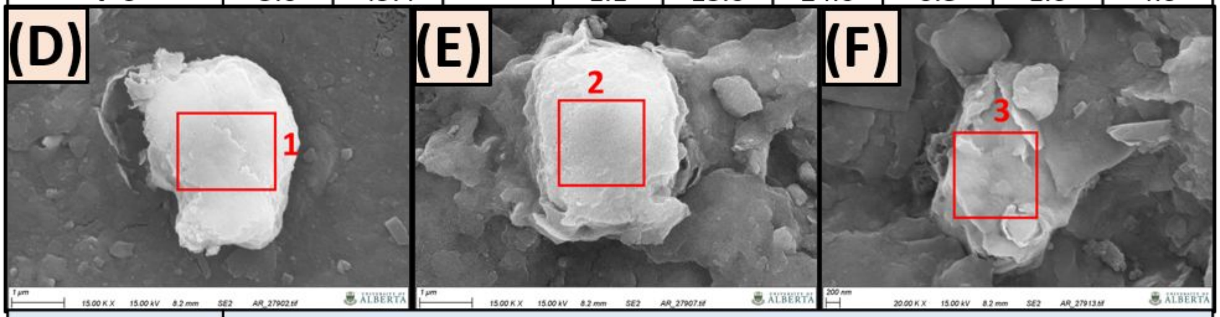

Spectrum	Elemental Composition – EDX (%)								
	C	O	Na	Mg	Al	Si	K	Ca	Fe
1-d	8.5	39.8		0.8	3.2	2.4		2.3	43.0
2-e	10.1	33.7	0.3	0.6	1.8	3.3	0.2	1.7	39.4
3-f	8.9	39.4	0.3	0.8	5.0	10.2	0.6	1.3	33.4

Figure S5. SEM-EDX of selected particle from selected sites. (A,B,C) represent particles collected from ARSW midQ, (D,E,F) are particles collected from HR hiQ.

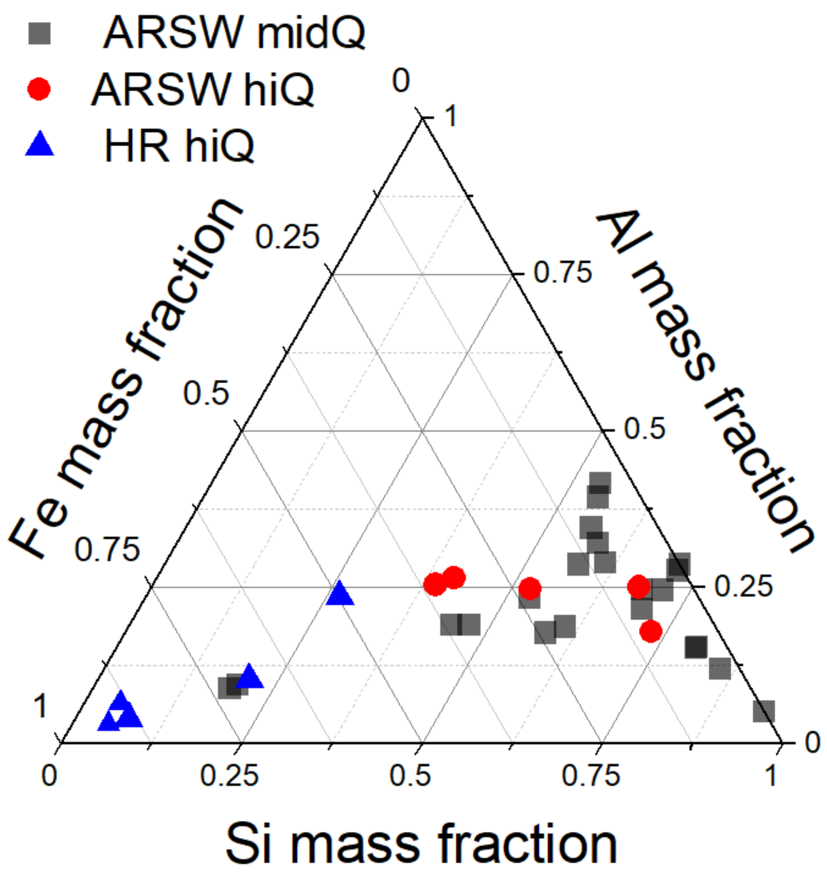

Figure S6. Ternary diagram of Al, Si, Fe mass fraction as determined by SEM-EDX. The mass fraction for selected particles in the SEM images were determined and the relative mass fractions of Al, Si, and Fe were determined for particles from three selected sites (ARSW midQ, ARSW hiQ, HR hiQ)

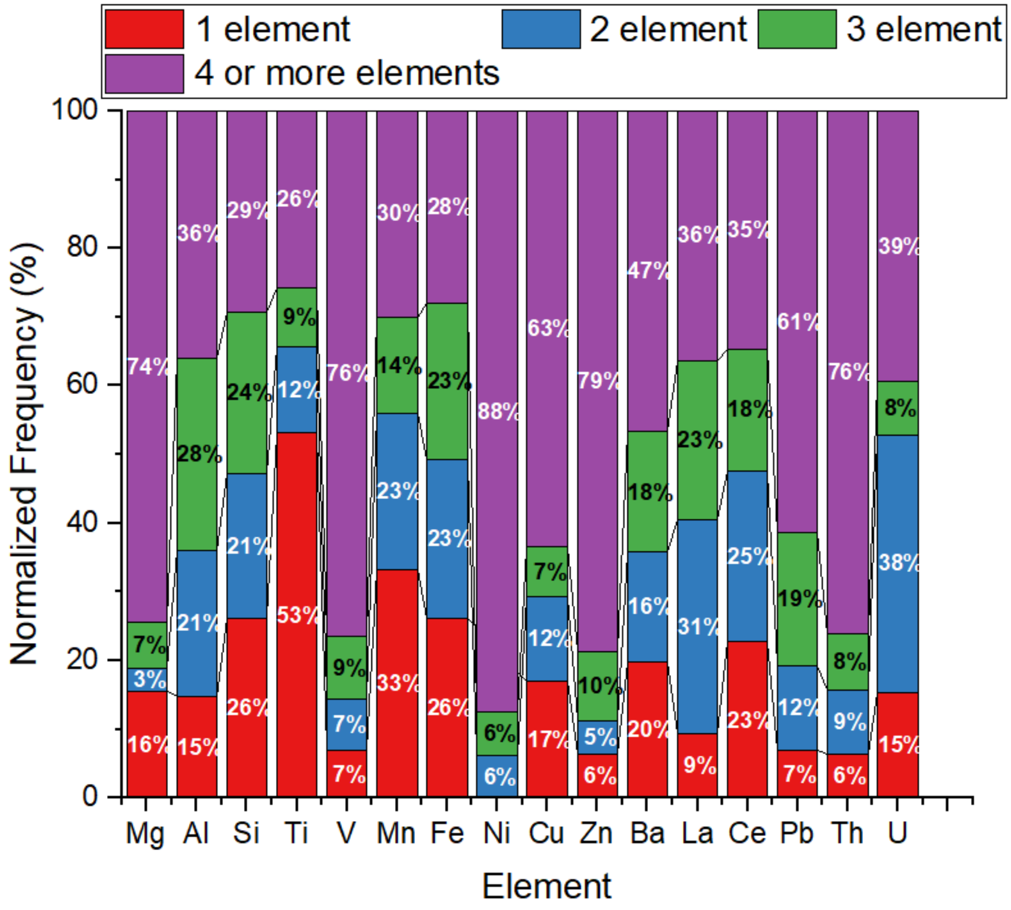

Figure S7. Proportion of single and multi-elements particles in ARSW midQ as grouped by element.

Table S2. Dissolved organic carbon concentrations for selected site used in this study

Site	Dissolved Organic Carbon (mg L^{-1})
Horse River (HR)	31.2
Horse River high flow (HR hiQ)	35.0
Clearwater River (CW)	12.7
Athabasca River low flow (ARSW lowQ)	12.4
Athabasca River middling flow (ARSW midQ)	11.8
Athabasca River high flow (ARSW hiQ)	11.7
Steepbank River (SB)	24.6

Table S3. Details of sampling sites at time of collection including field-measured water quality parameters.

Sample Number	Date	Weather	Location	Time	Total Depth (m)	Sample Depth (m)	Velocity (m/s)	Water Temperature (°C)	Pressure (mmHg)	DO (% L)	DO (mg L^{-1})	Conductivity (µS cm^{-1})	pH	ORP (mV)
HR	June 15, 2018	sunny, some wind	N 57.02099°<br>W 111.4801°	10:37 AM	1.3	0.6	0.49	13.9	736.2	106	10.56	127	7.78	221.3
HRhiQ	June 16, 2018	sun	N 56.719450°<br>W 111.398995°	11:56 AM	1.5	0.6	0.49	18.0	740.7	103	9.46	121.4	7.60	207.2
CW	June 17, 2018	sunny	N 56.73900°<br>W 111.37077°	9:01 AM	2.5	0.3	0.76	18.0	739.1	96.9	8.91	170.3	7.69	224.1
ARSW hiQ	June 17, 2018	sunny, warm	N 56.85718°<br>W 111.43113°	2:00 PM	2.3	0.3	0.91	18.9	737.7	105	9.42	190.6	7.55	175.50
ARSW midQ	June 18, 2018	sunny, warm, light breeze	N 56.85714°<br>W 111.43103°	9:35 AM	2.3	0.3	0.88	18.0	740.1	99	9.11	181	7.55	235.40
ARSW lowQ	June 26, 2018	cloudy, ~15°C, light breeze	N 56.85715°<br>W 111.43105°	9:18 AM	1.6	0.3	0.76	20.3	731.1	90.2	7.82	222.2	8.23	136.80
CW	June 26, 2018	cloudy	N 56.85715°<br>W 111.43105°	9:37 AM	1.6	1.2	0.64	20.3	731	90.2	7.81	222.7	8.22	127.20

Table S4. Total unfiltered metal concentrations for selected sites used in this study. All elements were quantified using a Thermo Fisher iCAP Qc with a helium collision gas (KED mode) as described in the methods.

Analyte	27Al	51V	55Mn	57Fe	59Co	60Ni	63Cu	66Zn	137Ba	139La	140Ce	208Pb	232Th	238U
units	mg/L	ug/L	ug/L	mg/L	ug/L	ug/L	ug/L	ug/L	ug/L	ug/L	ug/L	ug/L	ug/L	ug/L
LOD	0.000	0.0005	0.0003	0.000	0.00007	0.0008	0.0005	0.005	0.0004	0.00002	0.00003	0.00005	0.00002	0.00002
LOQ	0.000	0.002	0.002	0.000	0.0004	0.008	0.008	0.043	0.002	0.00011	0.0002	0.0003	0.00013	0.00009
**HR hiQ**	12.9	31.3	287	8.53	3.19	8.51	5.88	16.4	132	7.68	15.7	3.94	2.55	0.78
**HR**	3.79	9.43	129	3.20	1.05	3.85	2.34	5.14	54.2	2.39	4.70	1.32	0.76	0.37
**CW**	3.79	8.26	96.7	2.83	0.96	2.59	1.56	4.51	48.5	2.02	4.12	1.08	0.64	0.25
**ARSW lowQ**	9.8	23.5	148	5.81	2.31	7.11	5.16	12.9	169	5.36	10.3	2.98	1.67	1.03
**ARSW midQ**	47.8	113	489	29.2	9.35	26.6	20.2	57.3	508	21.5	43.9	12.5	7.29	2.64
**ARSW hiQ**	48.2	122	423	30.6	9.76	28.4	21.4	60.7	550	23.0	46.2	13.5	8.10	2.79
**SB**	1.35	3.35	66.8	1.36	0.45	1.37	0.72	1.92	38.0	0.78	1.59	0.44	0.25	0.13

Table S5. Total filtered (0.45 µm) metal concentrations for selected sites used in this study. All elements were quantified using a Thermo Fisher iCAP Qc with a helium collision gas (KED mode) as described in the methods (note concentration of Co, La, Ce, Pb, and Th are in ng/L).

Analyte	27Al	51V	55Mn	57Fe	59Co	60Ni	63Cu	66Zn	137Ba	139La	140Ce	208Pb	232Th	238U
units	ug/L	ug/L	ug/L	ug/L	ug/L	ug/L	ug/L	ug/L	ug/L	ug/L	ug/L	ug/L	ug/L	ug/L
LOD	0.03	0.002	0.0007	0.006	0.0003	0.002	0.0013	0.02	0.0007	0.000014	0.00006	0.00005	0.00003	0.00003
LOQ	0.16	0.007	0.004	0.04	0.0012	0.014	0.010	0.11	0.003	0.00007	0.0003	0.0003	0.00013	0.00013
**HR**	18.0	0.45		415	0.13	1.68	0.97	0.73	17.8	0.20	0.36	0.083	0.059	0.15
**HR hiQ**	30.2	0.46	45.8	412	0.28	1.96	1.23	1.01	17.5	0.25	0.55	0.10	0.085	0.14
**CW**	9.30	0.29	6.10	209	0.069	0.64	0.36	0.38	13.9	0.055	0.11	0.038	0.013	0.072
**ARSW lowQ**	12.6	0.34	4.31	45.0	0.085	1.60	1.26	0.54	45.8	0.040	0.073	0.024	0.011	0.38
**ARSW midQ**	17.7	0.38	7.65	94.4	0.10	2.31	2.14	0.53	40.3	0.13	0.25	0.067	0.039	0.47
**ARSW hiQ**	13.9	0.35	12.0	85.7	0.14	2.39	1.88	1.11	41.2	0.12	0.23	0.061	0.032	0.49
**SB**	15.4	0.22	17.6	267	0.10	0.54	0.22	0.55	20.0	0.054	0.10	0.022	0.017	0.052

Table S6. Operating conditions for Asymmetric Flow-field-flow fractionation and ICP-MS (total and dissolved metal concentrations).

Parameter	Setting
**AF4**	
Spacer	500 µm PTFE
Sample Loop volume (mL)	0.3
Injection flow rate (mL/min)	0.2
Crossflow rate (mL/min)	2.1 for 29 min, then linear decrease to 0 over 1 min
Channel flow rate (mL/min)	0.7
Focusing/elution time (min)	6 min / 44 min
**ICP-MS (Thermo Fisher iCAP Qc)**	
RF Power (W)	1550
Nebulizer gas flow (L/min)	1.03 (Argon)
Collision cell gas flow (mL/min)	5.0 (Helium)
Cooling gas flow (L/min)	14.0
Auxiliary gas flow rate (L/min)	0.8
Spray chamber	Cyclonic, Quartz (Thermo)
Sample and skimmer cone	Ni, with 2.8mm skimmer insert (Thermo)
Nebulizer	PFA-ST (Elemental Scientific)
Torch	Quartz torch w/ 2.0 mm quartz injector (Thermo)
Dwell times (ms)	0.1
Analytes	^{7}Li, ^{24}Mg, ^{27}Al, ^{51}V, ^{55}Mn, ^{56}Fe, ^{59}Co, ^{60}Ni, ^{63}Cu, ^{66}Zn, ^{75}As, ^{88}Sr, ^{89}Y, ^{95}Mo, ^{137}Ba, ^{208}Pb, ^{232}Th, ^{238}U
Indium tracer concentration (µg/L)	5

Table S7. Operating conditions for TOF-MS

**ICP-TOFMS (icpTOF 2R)**	
**Instrument Parameter**	**Value**
Nebulizer Gas Flow	0.85 – 1.0 ml/min
Sample Flow Rate	0.28 ml/min
Spray Chamber	Quartz cylconic
ICP RF Power	1550 W
Dwell time	3 ms
Transport efficiency	7-15%
Analytes	^{27}Al, ^{28}Si, ^{48}Ti, ^{55}Mn, ^{56}Fe, ^{57}Fe, ^{58}Ni, ^{60}Ni, ^{63}Cu, ^{64}Zn, ^{65}Cu, ^{66}Zn, ^{68}Zn, ^{110}Cd, ^{111}Cd, ^{112}Cd, ^{113}Cd, ^{114}Cd, ^{197}Au, ^{206}Pb, ^{207}Pb, ^{208}Pb
Analysis Time	60 s
Sample Flow Rate	0.3 ml/min

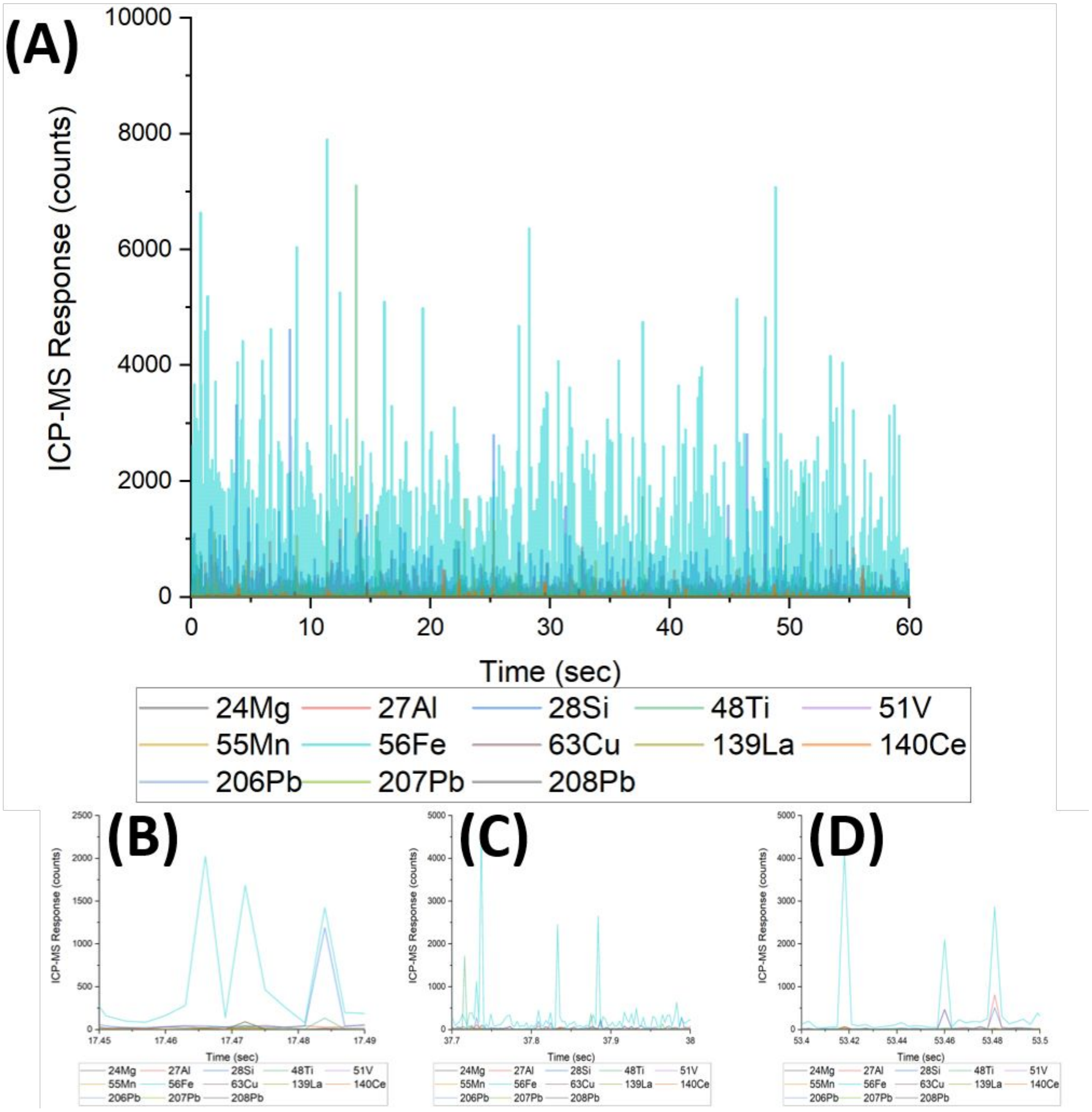

Figure S8. Raw data example from ARSW midQ. (A) Full time plot showing particulate signals from selected elements. (B,C,D) show select time intervals from (A) examining the spacing between particulate signals and the potential co-occurrence of elements within a signal. The size of the spikes is indicative of the mass of the particles. The noise that is evident in some the baseline of some elemental signal (specifically ^{56}Fe) could indicate either dissolved species or ubiquitous small particles below the detection limit.

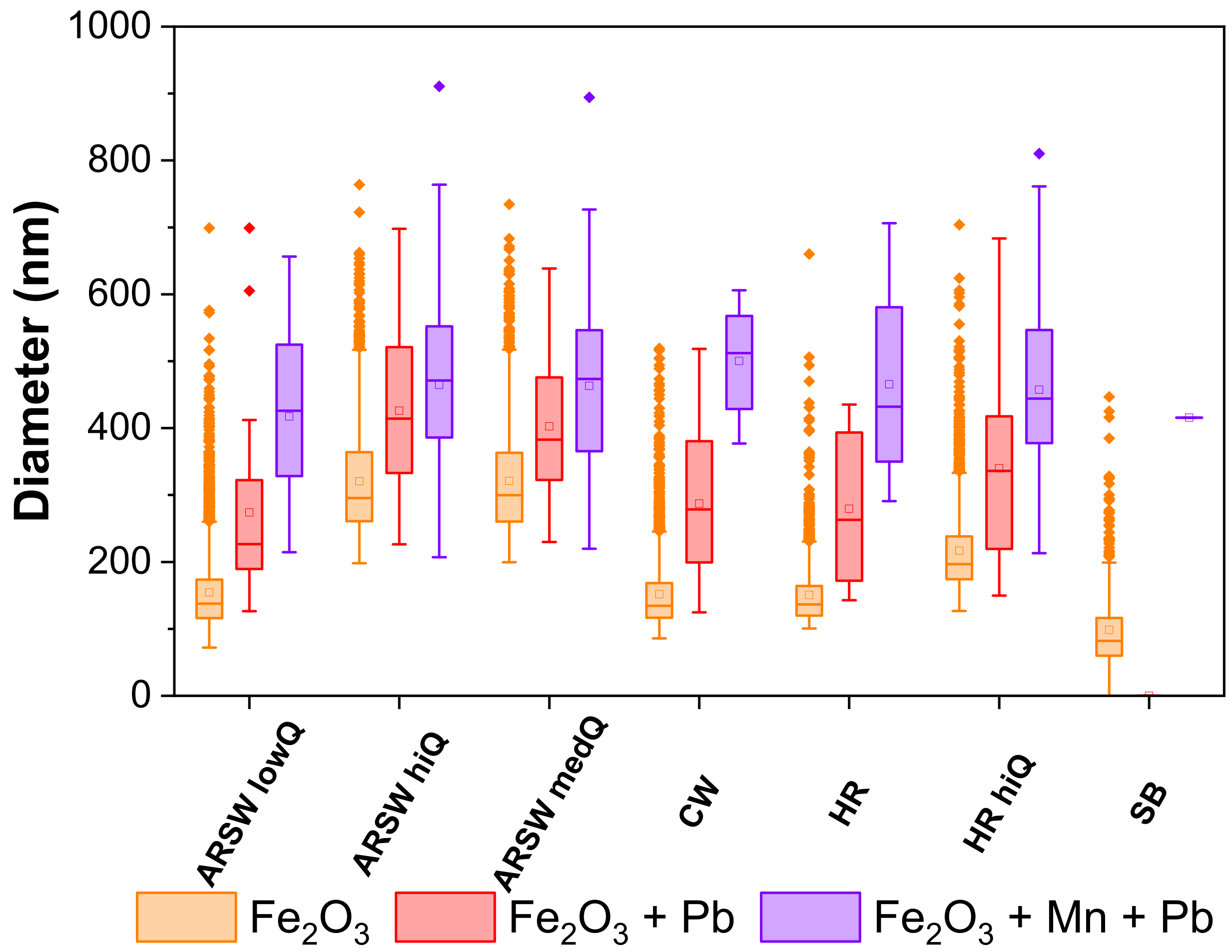

Figure S9. Calculated iron particulate sizes assuming a hematite density (ρ = 5.26 g cm^{-3}) and spherical geometry. Particle sizes are divided into three categories based on: 1) all particles containing iron, 2) those containing iron and lead, and 3) particles containing detectable masses of iron, lead, and manganese.
